# A *cis*-regulatory module activating transcription in the suspensor contains five *cis*-regulatory elements

**DOI:** 10.1007/s11103-015-0308-z

**Published:** 2015-03-22

**Authors:** Kelli F. Henry, Tomokazu Kawashima, Robert B. Goldberg

**Affiliations:** 1 Department of Molecular, Cell and Developmental Biology, University of California, Los Angeles, 610 Charles E. Young Dr. East, Los Angeles, CA 90095-7239 USA; 2 Present Address: Gregor Mendel Institute, Dr. Bohr-Gasse 3, 1030 Vienna, Austria

**Keywords:** *Phaseolus coccineus*, Scarlet runner bean, Suspensor, Gene regulatory network, *Cis*-regulatory elements, Promoter analysis

## Abstract

Little is known about the molecular mechanisms by which the embryo proper and suspensor of plant embryos activate specific gene sets shortly after fertilization. We analyzed the upstream region of the Scarlet Runner Bean (*Phaseolus coccineus*) *G564* gene in order to understand how genes are activated specifically in the suspensor during early embryo development. Previously, we showed that a 54-bp fragment of the *G564* upstream region is sufficient for suspensor transcription and contains at least three required *cis*-regulatory sequences, including the 10-bp motif (5′-GAAAAGCGAA-3′), the 10 bp-like motif (5′-GAAAAACGAA-3′), and Region 2 motif (partial sequence 5′-TTGGT-3′). Here, we use site-directed mutagenesis experiments in transgenic tobacco globular-stage embryos to identify two additional *cis*-regulatory elements within the 54-bp *cis*-regulatory module that are required for *G564* suspensor transcription: the Fifth motif (5′-GAGTTA-3′) and a third 10-bp-related sequence (5′-GAAAACCACA-3′). Further deletion of the 54-bp fragment revealed that a 47-bp fragment containing the five motifs (the 10-bp, 10-bp-like, 10-bp-related, Region 2 and Fifth motifs) is sufficient for suspensor transcription, and represents a *cis*-regulatory module. A consensus sequence for each type of motif was determined by comparing motif sequences shown to activate suspensor transcription. Phylogenetic analyses suggest that the regulation of *G564* is evolutionarily conserved. A homologous *cis*-regulatory module was found upstream of the *G564* ortholog in the Common Bean (*Phaseolus vulgaris*), indicating that the regulation of *G564* is evolutionarily conserved in closely related bean species.

**Electronic supplementary material:**

The online version of this article (doi:10.1007/s11103-015-0308-z) contains supplementary material, which is available to authorized users.

## Introduction

In most higher plants, the zygote divides asymmetrically to form a small apical cell and a large basal cell with distinct developmental fates (Goldberg et al. 1994). The apical cell differentiates into the embryo proper, which will become the next generation plant; whereas, the basal cell generates the hypophysis and suspensor. The hypophysis will be incorporated into the root meristem of the developing embryo proper (Dolan et al. 1993). The suspensor is a terminally differentiated embryonic region that physically connects the embryo proper to the maternal tissues and transports nutrients and growth regulators to the embryo proper before degenerating later during embryogenesis (Yeung and Meinke 1993).

The precise mechanisms that determine the developmental fates of the apical and basal cells remain unknown. A number of studies using individual genes and whole-genome transcriptome profiling have shown that different gene sets are activated in the globular-stage embryo proper and suspensor shortly after fertilization (Weterings et al. 2001; Haecker et al. 2004; Le et al. 2007; Ueda et al. 2011; Belmonte et al. 2013; Slane et al. 2014). Little is known, however, about the *cis*-regulatory sequences and *trans*-acting factors that activate genes in different embryonic regions. Nor is it known how these genes are connected into regulatory networks that control their expression in precise spatial and temporal patterns during embryonic development (Peter and Davidson 2015).

**Fig. 1:**
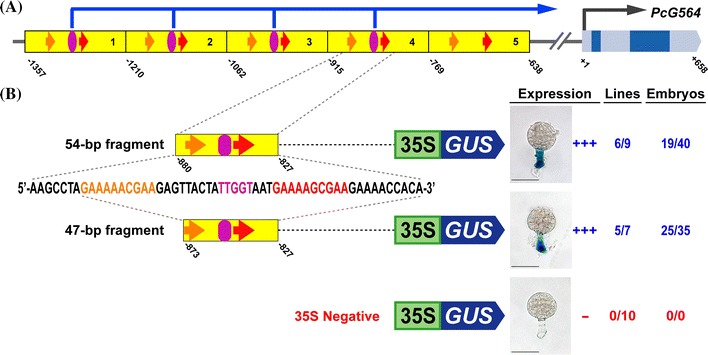
A 47-bp region contains all of the sequences required for suspensor transcription. **a** Conceptual representation of the *G564* gene and upstream region. **b** Suspensor GUS activity in transgenic globular-stage tobacco embryos containing GOF fragments from the *G564* upstream region.* Names* and* conceptual* representations of the constructs are to the left of each embryo. *Numbers* indicate positions relative to the *G564* transcription start site (+1) (Weterings et al. 2001). *Yellow boxes* indicate the 150-bp promoter repeats (Weterings et al. 2001; Kawashima et al. 2009). *Red arrows, orange arrows* and *purple ovals* indicate the 10-bp, 10-bp-like, and Region 2 motifs, respectively (Kawashima et al. 2009). *35S/GUS* indicates the *CaMV 35S* minimal promoter/*GUS* gene. The sequences of the 10-bp, 10-bp-like and Region 2 motifs were taken from Kawashima et al. (2009) and are shown in *red, orange,* and *purple fonts*, respectively. +++ in the Expression column indicates that suspensor GUS activity was strong and detected by 2-h incubation for a majority of the GUS-positive lines; − in the Expression column indicates no detectable suspensor GUS activity. *Numbers in the Lines column* indicate the number of individual transformants displaying suspensor GUS activity over the total number of individual transformants analyzed. *Numbers in the Embryos column* indicate the number of embryos displaying suspensor GUS activity by 24-h incubation over the total number of analyzed embryos of GUS-positive lines. Photographs were taken after 24-h GUS incubation (Scale bar: 50 μm)

Previously, we used the giant embryos of the Scarlet Runner Bean, *Phaseolus coccineus*, to identify suspensor-specific mRNAs (Weterings et al. 2001; Le et al. 2007; Kawashima and Goldberg 2010). These embryos contain a large, highly specialized suspensor that is particularly suited for region-specific gene expression studies early in embryo development (Le et al. 2007; Kawashima and Goldberg 2010; Henry and Goldberg 2015). One Scarlet Runner Bean suspensor-specific mRNA, designated as G564, which encodes a protein of unknown function, first appears in the basal cells of the four-cell embryo, and then accumulates to a high level in the suspensor at later developmental stages (Weterings et al. 2001). The spatial expression pattern of the *G564* gene is controlled primarily at the transcriptional level by sequences in its proximal upstream region (Weterings et al. 2001; Kawashima et al. 2009). The *G564* upstream region contains a ~150-bp sequence repeated five times in tandem **(**Fig. 1a) (Weterings et al. 2001; Kawashima et al. 2009). We dissected the fourth 150-bp repeat and found that it contains a 54-bp positive *cis*-regulatory module that is sufficient to program suspensor transcription shortly after fertilization, and contains at least three suspensor *cis*-regulatory elements designated as the (1) 10-bp motif (5′-GAAAAGCGAA-3′), (2) 10-bp-like motif (5′-GAAAAACGAA-3′), and (3) Region 2 motif (5′-TTGGT-3′) **(**Fig. 1b) (Kawashima et al. 2009). The *G564* 54-bp suspensor *cis*-regulatory module is conserved in all five 150-bp repeats, with some sequence differences (Kawashima et al. 2009). We showed that the machinery that activates suspensor transcription using these motifs is conserved among flowering plants (Kawashima et al. 2009).

In this paper, we present experiments identifying two additional *cis*-regulatory elements within the 54-bp *cis*-regulatory module that are required for *G564* suspensor transcription. Mutagenesis of the 54-bp fragment identified a new suspensor *cis*-regulatory element, the Fifth motif (5′-GAGTTA-3′), and an additional 10-bp sequence, designated the 10-bp-related motif (5′-GAAAACCACA-3′), that are required for suspensor transcription. Further deletion of the 54-bp fragment revealed that a 47-bp fragment containing only the five motifs (the 10-bp, 10-bp-like, 10-bp-related, Region 2 and Fifth motifs) is sufficient for suspensor transcription. We also show that the Scarlet Runner Bean *G564* suspensor *cis*-regulatory module is conserved in the *G564* ortholog (*PvG564*) of the Common Bean, *Phaseolus vulgaris,* suggesting that these motifs program *G564* expression patterns in the giant suspensors of two closely-related bean species. Together, our results have identified five suspensor *cis*-regulatory elements, three of which are functionally equivalent, that activate *G564* transcription specifically in the suspensor shortly after fertilization.

## Materials and methods

### Generating gain-of-function (GOF) and site-directed mutagenesis constructs

For GOF and mutagenesis analyses of the *G564* upstream region, the GOF12 vector described by Kawashima et al. (2009), which contains the *G564* 54-bp fragment (−880 to −827) upstream of the *CaMV* *35S* minimal promoter (Benfey et al. 1990; Koltunow et al. 1990) and the *β*-*glucuronidase (GUS)* reporter gene (Jefferson et al. 1987), was used as a PCR template with primers containing the desired mutation and EcoRI or BamHI restriction sites (Kawashima et al. 2009). Primer sequences used for mutagenesis are listed in Online Resource 1. The amplified DNA fragments were then digested with EcoRI and BamHI and ligated into the EcoRI- and BamHI-digested GOF12 vector. *G564* fragment regions in the mutagenesis constructs were sequenced to confirm that they contained the correct mutated bases.

### Plant transformation

Tobacco (*Nicotiana tabacum* cv SR1) plants were transformed and regenerated by using the leaf disk procedure (Horsch et al. 1985). At least six independent transformants were generated for each construct. The promoter/*GUS* region from each individual transformant was sequenced to ensure that there were no rearrangements. A total of 16 different mutagenesis constructs and 129 individual tobacco transformants were generated in order to carry out this study.

### GUS histochemical assay

Transgenic tobacco seeds were harvested 8 days after pollination (DAP). Globular-stage embryos were hand-dissected from seeds and assayed for GUS activity after 2 and 24 h at 37 °C, as described previously (Kawashima et al. 2009). Embryos were photographed under bright-field illumination using a compound microscope (LEICA 5000B). T1 seeds from GUS-negative lines were tested for kanamycin-resistant segregation after selfing to confirm that the T-DNA was not silenced. In total, approximately 800 individual globular-stage embryos were assayed for GUS activity in order to generate the results reported in this study.

## Results

### A 47-bp fragment contains all of the sequences required for suspensor transcription

We used the Scarlet Runner Bean *G564* gene to identify *cis*-regulatory elements required for suspensor transcription. Previously, we showed that a gain-of-function (GOF) construct containing a 54-bp fragment of the *G564* upstream region can program suspensor-specific transcription when fused to a *Cauliflower Mosaic Virus* (*CaMV*) *35S* minimal promoter/*GUS* vector and introduced into tobacco plants (Kawashima et al. 2009). This 54-bp fragment contains the previously identified 10-bp motif (5′-GAAAAGCGAA-3′), 10-bp-like motif (5′-GAAAAACGAA-3′), and Region 2 motif that contains the sequence 5′-TTGGT-3′ **(**Fig. 1b) (Kawashima et al. 2009). To identify the minimal sequence required for *G564* suspensor transcription, we deleted the distal 7-bp of the 54-bp fragment, and showed that it produced strong suspensor GUS activity under the direction of the *CaMV* *35S* minimal promoter in transgenic tobacco embryos, similar to that observed for the 54-bp fragment **(**Fig. 1b). By contrast, a *CaMV 35S* minimal promoter/*GUS* construct produced no detectable suspensor GUS activity. Thus, the 47-bp fragment is the minimal *cis*-regulatory module that can activate *G564* suspensor transcription.

### A third 10-bp sequence is required for suspensor transcription

**Fig. 2:**
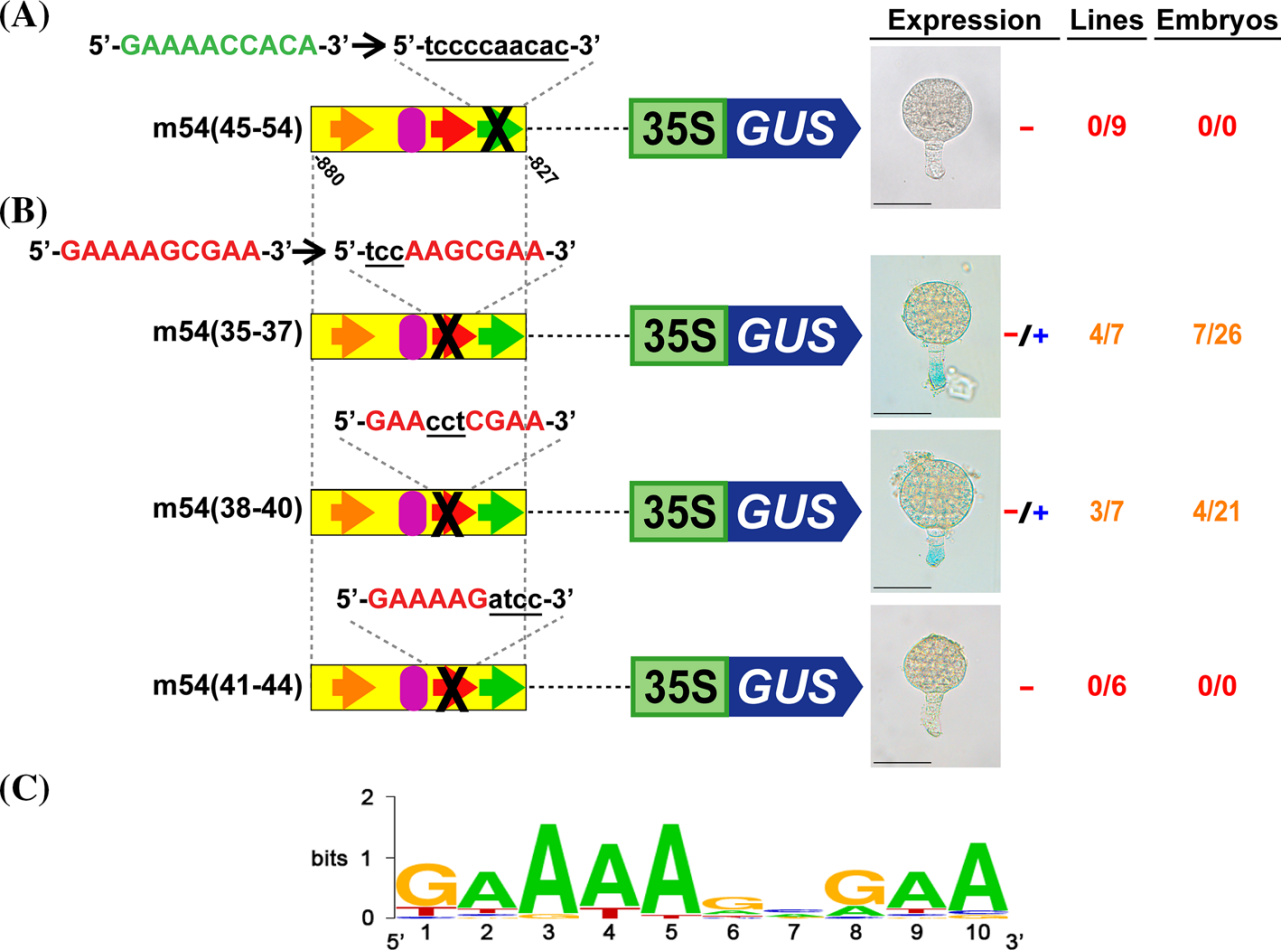
A third 10-bp motif is required for suspensor transcription. **a** and **b** Suspensor GUS activity in transgenic tobacco embryos containing 54-bp GOF constructs with mutations within the 10-bp-related motif (**a)** or 10-bp motif **(b)**. **c** A consensus 10-bp/10-bp-like/10-bp-related motif sequence was generated from DNA sequences shown to function as the 10-bp motif in transgenic tobacco embryos (Kawashima et al. 2009). *Green arrows* indicate the 10-bp-related motif. The sequences of the 10-bp and 10-bp-related motifs are shown in *red* and *green fonts*, respectively. *Black crosses* indicate mutations in these motifs. The mutation sequence is written in *black*, *lower case*, and *underlined font*. +++ in the Expression column indicates that suspensor GUS activity was strong and detected by 2-h incubation for a majority of the GUS-positive lines; −/+ in the Expression column indicates that suspensor GUS activity was weak and not detected by 2-h incubation in the majority of embryos from GUS-positive lines; − in the Expression column indicates no detectable suspensor GUS activity. *Numbers in the Lines column* indicate the number of individual transformants displaying suspensor GUS activity over the total number of individual transformants analyzed. *Numbers in the Embryos column* indicate the number of embryos displaying suspensor GUS activity by 24-h incubation over the total number of analyzed embryos of GUS-positive lines. Photographs were taken after 24-h GUS incubation (Scale bar: 50 μm)

Within the 54-bp *G564* fragment, there is 10-bp (−836/−827) between the 10-bp motif and the 3′ end of the fragment, in which there is enough space for another suspensor *cis*-regulatory element **(**Fig. 1b). To identify additional suspensor *cis*-regulatory sequences, we generated GOF constructs in which mutated versions of the 54-bp fragment were fused to the *CaMV* *35S* minimal promoter/*GUS* vector, and introduced these constructs into tobacco plants. A GOF construct in which the 10-bp sequence at −836/−827 (5′-GAAAACCACA-3′) was mutated [m54(45–54)] showed no GUS activity, indicating that there is an additional motif that is required for suspensor transcription **(**Fig. 2a). Because this 10-bp sequence at −836/−827 is similar to the 10-bp and 10-bp-like motifs, differing by 3-bp, we have designated it as the 10-bp-related motif. Therefore, three 10-bp sequences in the 54-bp fragment are required for suspensor transcription: the 10-bp (−846/−837), 10-bp-like (−873/−864) and 10-bp-related (−836/−827) motifs.

To determine the core sequence of the 10-bp/10-bp-like/10-bp-related motifs, we took the 10-bp motif as a representative of these three motifs and mutated the first 3-bp, the second 3-bp and the last 4-bp of the 10-bp motif within the 54-bp fragment **(**Fig. 2b). Although the 10-bp motif is known to tolerate a 3-bp mismatch without affecting the level of suspensor transcription (Kawashima et al. 2009), we hypothesized that the mismatches would not be tolerated if they were all clustered together, rather than being spread throughout the motif. GUS activity in the suspensor was significantly decreased when the first and second 3-bp were mutated [m54(35–37) and m54(38–40)] and completely abolished when the last 4-bp were mutated [m54(41–44)] **(**Fig. 2b). These results demonstrated that at least one nucleotide in each of these three regions is critical for the function of the 10-bp motif.

Previously, we generated a consensus sequence for the 10-bp motif from divergent 10-bp sequences known to function as the 10-bp motif **(**Fig. 2c) (Kawashima et al. 2009). Each position of the 10-bp motif can tolerate a mismatch without affecting the level of suspensor transcription. All of the functional 10-bp sequences contain up to three mismatches relative to the 10-bp motif sequence of 5′-GAAAAGCGAA-3′ (Kawashima et al. 2009), and this sequence represents the nucleotide most commonly found in each position of the motif for all of the sequences that function as the 10-bp motif. Figure 2b shows that three mismatches cannot be tolerated if the mismatches are clustered in one part of the 10-bp motif. Taken together, we determined a consensus for the 10-bp/10-bp-like/10-bp-related motifs of 5′-GAAAAGCGAA-3′ with up to three non-adjacent mismatches.

### The complete sequence of the Region 2 motif was identified

**Fig. 3:**
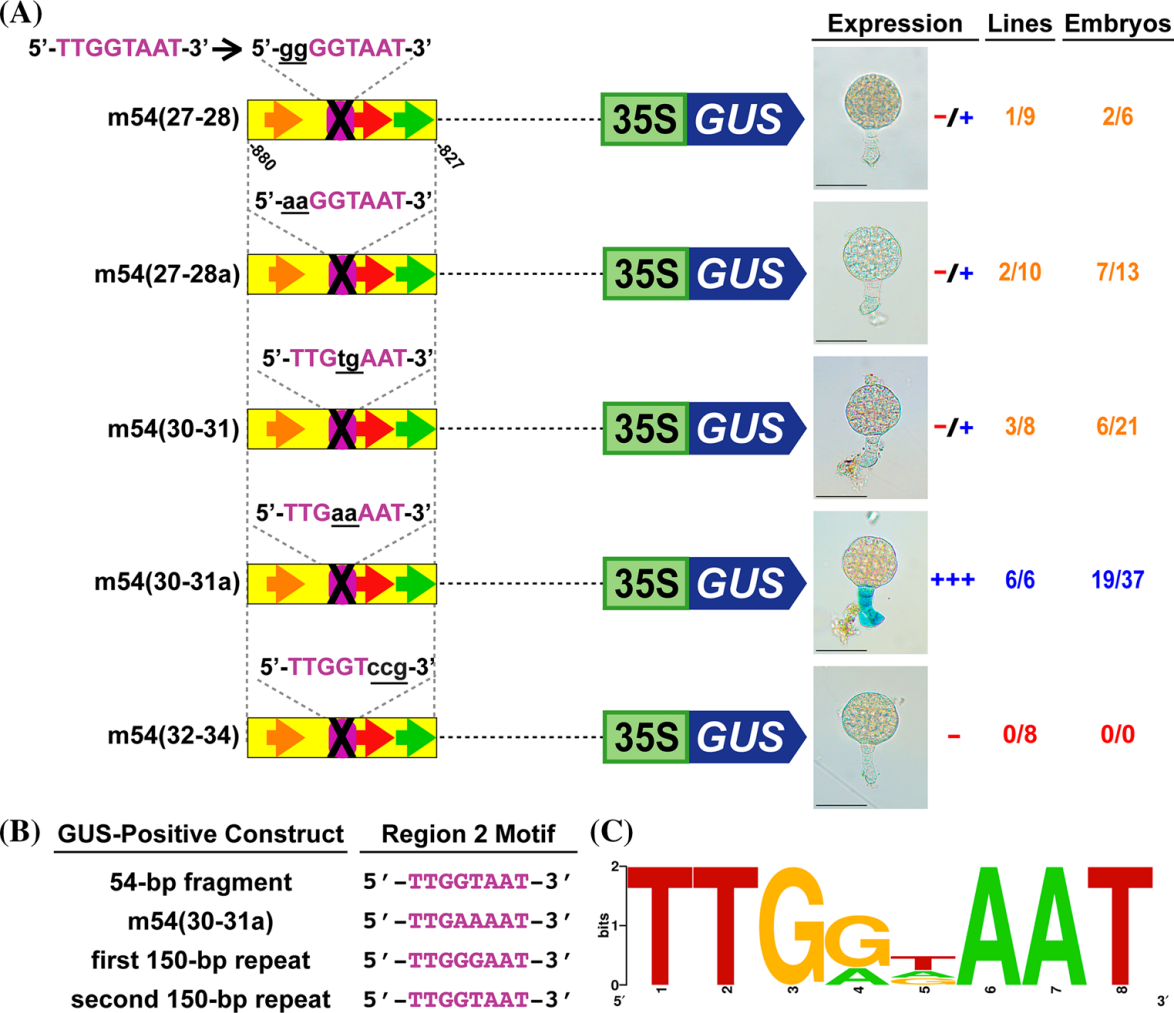
A consensus sequence for the Region 2 motif. **a** Suspensor GUS activity in transgenic tobacco embryos containing 54-bp GOF constructs with mutations within the Region 2 motif. **b** Sequences of the Region 2 motif in GOF constructs that activate suspensor transcription. Suspensor GUS activity in transgenic tobacco embryos containing the first and second 150-bp repeats is presented in Kawashima et al. (2009). **c** A consensus sequence for the Region 2 motif was created by WebLogo (Crooks et al. 2004) using Region 2 motif sequences shown in **b** that function within the suspensor. *Purple ovals* indicate the Region 2 motif. Region 2 motif sequences are shown in *purple font*. *Black crosses* indicate mutations in this motif. The mutation sequence is written in *black*, *lower case,* and *underlined font*. Other figure details are the same as those outlined in Fig. 1 and 2. Photographs were taken after 24-h GUS incubation (Scale bar: 50 μm)

We previously identified the partial Region 2 motif sequence as 5′-TTGGT-3′ through sequence homology between the 150-bp *G564* GUS-positive repeats (first, second and fourth repeats) and the GUS-negative fifth repeat **(**Fig. 1a) (Kawashima et al. 2009). The Region 2 motif was shown to be functional in the 54-bp fragment by mutating the central guanine at position 29 of the 54-bp fragment to adenine, rendering it inactive (Kawashima et al. 2009). In order to functionally test which nucleotides in Region 2 are required for suspensor transcription, we generated mutations across the Region 2 motif either 2-bp or 3-bp at a time and transferred GOF constructs containing these mutant motifs into transgenic tobacco plants **(**Fig. 3a). Transversional mutagenesis was used to impose the biggest change on the sequence; however transversional mutations of this GT-rich motif resulted in another GT-rich sequence. Consequently, constructs were also made using adenine substitutions **(**Fig. 3a).

Mutation of the 5′ TT using either mutagenesis strategy [m54(27–28) and m54(27–28a)] resulted in significantly decreased suspensor GUS activity. Mutation of the central GT to TG [m54(30–31)] also resulted in significantly decreased suspensor GUS activity. By contrast, mutation of GT to AA [m54(30–31a)], creating the sequence 5′-TTGAAAAT-3′, did not alter GUS activity. Finally, mutation of the 3′ AAT [m54(32–34)] abolished GUS activity, demonstrating that the Region 2 motif is longer than previously predicted. Taken together, these results show that the important nucleotides in the Region 2 motif are 5′-TTGGTAAT-3′, and that 5′-TTGAAAAT-3′ can also function as the Region 2 motif **(**Fig. 3a).

We identified a consensus for the Region 2 motif by comparing Region 2 motif sequences within all 150-bp *G564* repeats that we showed previously can drive *GUS* gene expression in the suspensor of transgenic tobacco embryos (first, second and fourth repeats) (Kawashima et al. 2009). No other sequence element(s) in the 150-bp repeats can compensate for the function of the Region 2 motif, as was shown by a natural point mutation in the fifth repeat that causes a loss of suspensor GUS activity **(**Fig. 1a) (Kawashima et al. 2009). The sequence of the Region 2 motif in the second repeat and the 54-bp fragment within the fourth repeat is 5′-TTGGTAAT-3′, whereas the Region 2 sequence in the first repeat is 5′-TTGGGAAT-3′ **(**Fig. 3b). We combined this information with the knowledge that 5′-TTGAAAAT-3′ can also function as the Region 2 motif **(**Fig. 3a), generating a sequence logo of 5′-TTG(A/G)(A/G/T)AAT-3′ for the Region 2 motif **(**Fig. 3c). This consensus sequence is representative of the Region 2 motif in each 150-bp *G564* upstream repeat, with the exception of the fifth repeat.

### A fifth motif is required for suspensor transcription

**Fig. 4:**
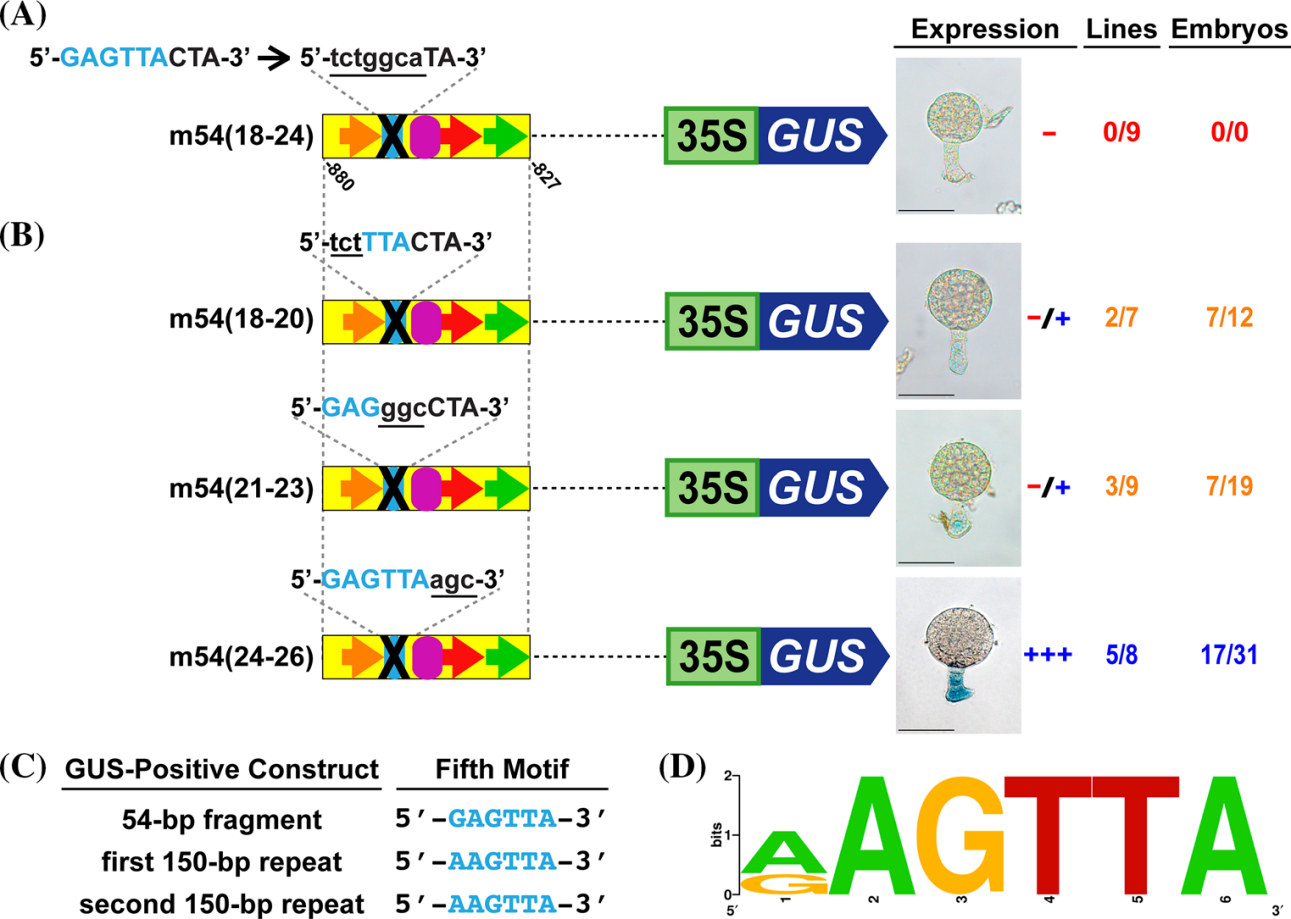
A fifth motif is required for suspensor transcription. **a** and **b** Suspensor GUS activity in transgenic tobacco embryos containing 54-bp GOF constructs with mutations within the Fifth motif region. **c** Sequences of the Fifth motif in GOF constructs that activate suspensor transcription. Suspensor GUS activity in transgenic tobacco embryos containing the first and second 150-bp repeats is presented in Kawashima et al. (2009). **d** A consensus sequence for the Fifth motif was created by WebLogo (Crooks et al. 2004) using Fifth motif sequences shown in **c** that function within the suspensor. *Blue ovals* indicate the Fifth motif. The sequences of the Fifth motif are shown in *blue font*. *Black crosses* indicate mutations in this motif. The mutation sequence is written in *black*, *lower case*, and *underlined font*. Other figure details are the same as those outlined in Figs. 1, 2, 3. Photographs were taken after 24-h GUS incubation (Scale bar: 50 μm)

Within the 54-bp fragment there is 9-bp between the 10-bp-like motif and Region 2 motif—providing enough space for a fifth *cis*-regulatory element **(**Fig. 1b). To determine whether there is a fifth suspensor *cis*-regulatory element, we mutated the sequence 5′-GAGTTAC-3′ in the region between the 10-bp-like and Region 2 motifs in the 54-bp fragment **(**Fig. 4a). Mutation of 5′-GAGTTAC-3′ [m54(18–24)] abolished suspensor GUS activity completely, indicating the presence of a fifth DNA control element required for suspensor transcription.

To define the length of the Fifth motif, we generated constructs by mutating the 9-bp between the 10-bp-like motif and Region 2 motif 3-bp at time **(**Fig. 4b). Mutation of 5′-GAG-3′ [m54(18–20)] and 5′-TTA-3′ [m54(21–23)] caused a significant decrease in GUS activity, indicating that these sequences are part of the Fifth motif. However, mutation of the next 3-bp, 5′-CTA-3′ [m54(24–26)], had no effect on GUS activity. Therefore, the final cytosine mutated in the m54(18–24) construct was not required for suspensor transcription. Taken together, these results showed that the important nucleotides in the Fifth motif are 5′-GAGTTA-3′.

We determined a consensus sequence for the Fifth motif through comparison of the Fifth motif sequence in GUS-positive 150-bp repeats (first, second and fourth) **(**Fig. 4c) (Kawashima et al. 2009). No other sequence in the 150-bp repeats closely resembles the Fifth motif, so we assume that the sequences in the position of the Fifth motif in the first and second repeats are functional. The sequence of the Fifth motif in the 54-bp fragment within the fourth repeat is 5′-GAGTTA-3′. The sequence in the position of the Fifth motif in the first and second repeats is 5′-AAGTTA-3′. Based on these results, we constructed a sequence logo for the Fifth motif with a consensus sequence of 5′-(A/G)AGTTA-3′ **(**Fig. 4d).

### The 150-bp tandem repeats and suspensor motifs are conserved in the Common Bean *G564* upstream region

**Fig. 5:**
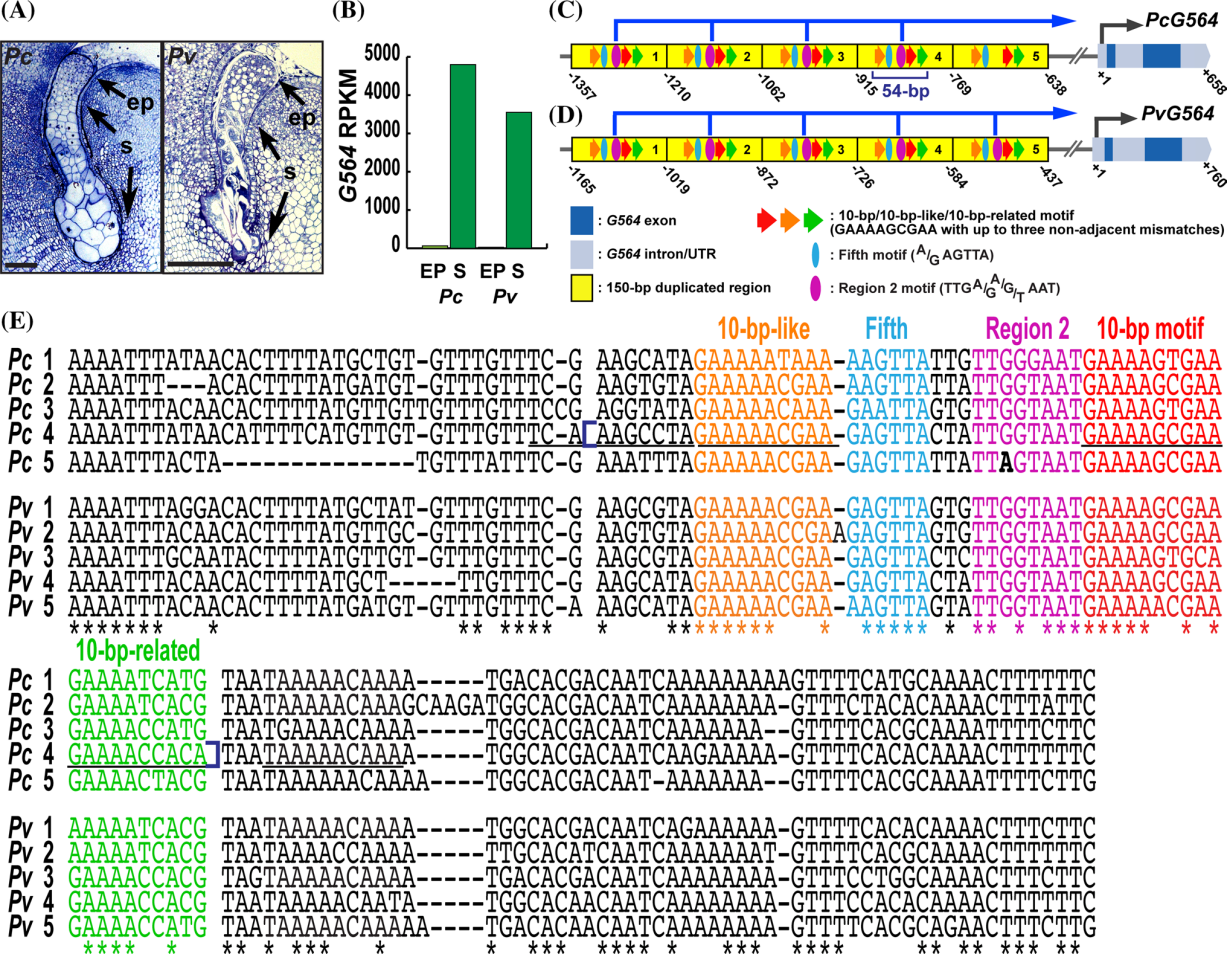
The *G564* gene and mRNA abundance in Scarlet Runner Bean and Common Bean. **a** Plastic section of Scarlet Runner Bean [*Phaseolus coccineus* (*Pc*)] globular-stage embryo and paraffin section of Common Bean [*Phaseolus vulgaris* (*Pv*)] globular-stage embryo. **b** Scarlet Runner Bean and Common Bean G564 mRNA prevalences determined by RNA-Seq analysis of laser-captured globular-stage embryo proper and suspensor regions. **c** and **d** Conceptual representations of the *G564* gene and upstream region in Scarlet Runner Bean **(c)** and Common Bean **(d)**. *Dark blue boxes* represent exons. *Light blue boxes* represent introns and UTRs. *Yellow boxes* represent 150-bp tandem repeats in the upstream region (Weterings et al. 2001; Kawashima et al. 2009). *Red, orange*, and* green arrows; purple ovals;* and* blue ovals* indicate the 10-bp, 10-bp-like and 10-bp-related motifs; Region 2 motif; and Fifth motif, respectively. *Dark blue* bracket designates the 54-bp region that was analyzed in this study. *Numbers* indicate positions relative to the transcription start site (+1). **e** Nucleotide sequence alignment of the five *G564* 150-bp tandem repeats. Nucleotides conserved across all five *G564* 150-bp repeats from Scarlet Runner Bean and Common Bean are indicated by *asterisks*. The 10-bp, 10-bp-like and 10-bp-related motifs, Region 2 motif and Fifth motif are shown in *red, orange, green, purple* and *blue font*, respectively. The bold A in the *PcG564* fifth 150-bp repeat is a natural mutation that makes the Region 2 motif non-functional (Kawashima et al. 2009). Sequences shown to function as the 10-bp motif are underlined (Kawashima et al. 2009). The number to the left of the aligned sequences indicates the position of each 150-bp repeat. Gaps were introduced for optimal alignment. ep, embryo proper; *Pc*, *Phaseolus coccineus*; *Pv*, *Phaseolus vulgaris*; RPKM, reads per kilobase per million; *s*, suspensor

The recent release of the Common Bean (*Phaseolus vulgaris*) genome (Schmutz et al. 2014) provides an opportunity to compare *G564* expression and promoter sequences in two related bean species with giant suspensors **(**Fig. 5a) (Henry and Goldberg 2015) that diverged ~2 million years ago (mya) (Delgado-Salinas et al. 2006). We used Illumina sequencing technology to profile the mRNA populations of laser-capture microdissected embryo proper and suspensor regions from Scarlet Runner Bean and Common Bean globular-stage embryos, and mapped RNA-Seq reads to the sequenced Common Bean genome (GEO accession GSE57537). Common Bean G564 mRNA was up-regulated ~140-fold in the globular-stage suspensor relative to the embryo proper, similar to what we observed for Scarlet Runner Bean **(**Fig. 5b) (Weterings et al. 2001; Henry and Goldberg 2015). We compared the *G564* upstream region in Scarlet Runner Bean and Common Bean, and found that the Common Bean *G564* promoter also contains the five 150-bp tandem repeats **(**Fig. 5c–e). These tandem promoter repeats are not found in the upstream region of *G564*-related genes in soybean or *Arabidopsis*, which diverged from the Scarlet Runner Bean 19 and 120 mya, respectively (Lavin et al. 2005). Therefore, the 150-bp repeats originated before Scarlet Runner Bean and Common Bean diverged from their common ancestor, but after the divergence of these bean species from their soybean (*Glycine max*) relative within the Legume family.

Sequences nearly identical to the 10-bp, 10-bp-like, 10-bp-related, Region 2 and Fifth motifs were found in each Common Bean 150-bp repeat **(**Fig. 5e). The one exception was the functional Region 2 motif sequence in the Common Bean fifth repeat, in contrast with the natural mutation in the Scarlet Runner Bean fifth repeat Region 2 sequence (see bold A in Fig. 5e) that makes it non-functional (Kawashima et al. 2009). The suspensor motifs identified in the Common Bean *G564* upstream region are most likely functional because the *G564* promoter sequences and suspensor-specific expression patterns are nearly indistinguishable in both species (Fig. 5b, e).

## Discussion

### Five positive *cis*-regulatory elements are required to activate *G564* suspensor transcription

We used the Scarlet Runner Bean *G564* gene to identify *cis*-regulatory elements required for activating suspensor transcription shortly after fertilization. A 54-bp fragment within the fourth 150-bp repeat in the upstream region of *G564* is sufficient for suspensor-specific transcription and contains three previously identified suspensor *cis*-regulatory elements: the 10-bp motif, 10-bp-like motif and the Region 2 motif (Kawashima et al. 2009). Here, we used site-directed mutagenesis of the 54-bp fragment to identify a new *cis*-regulatory element, the Fifth motif, which is required for suspensor-specific transcription of *G564* **(**Fig. 4a). We also identified a third 10-bp motif, which is required for suspensor transcription **(**Fig. 2a). Although the five suspensor *cis*-regulatory elements are densely packed in the *G564* suspensor module, these five motifs constitute discrete *cis*-regulatory elements. The Fifth motif and Region 2 motif are clearly separated by 3 bp **(**Fig. 5e). Previously, we showed that the Region 2 motif and the 10-bp motif are discrete *cis*-regulatory elements that can function even when the spacing and orientation are altered (Kawashima et al. 2009).

### Positioning of the *cis*-regulatory elements is flexible

A 47-bp fragment of the *G564* upstream region is sufficient for suspensor transcription, and contains the 10-bp motif, 10-bp-like motif, 10-bp-related motif, Region 2 motif and Fifth motif. The 47-bp fragment is the minimal *cis*-regulatory module that can activate suspensor transcription shortly after fertilization **(**Fig. 1b), and a change in almost any sequence within this module disrupts suspensor-specific transcription (Fig. 2–4) (Kawashima et al. 2009). The tight arrangement of *cis*-regulatory elements in the 47-bp suspensor module initially suggested that this module exemplifies the “enhanceosome” model of *cis*-regulatory module organization, in which the precise arrangement of *cis*-regulatory elements is essential for promoting cooperative interactions among bound transcription factors (Spitz and Furlong 2012). However, recent *cis*-regulatory analysis of the Scarlet Runner Bean *GA 20*-*oxidase* gene upstream region showed that these same motifs are required for suspensor transcription of *GA 20*-*oxidase* even though they have a different order, orientation and spacing (Henry 2014). It is just the presence of the motifs, not their relative positions, that is essential for function, similar to the *Drosophila* *eve* stripe two and *sog* *cis*-regulatory modules (Peter and Davidson 2015). This suggests that the organization of control elements within the *G564* suspensor *cis*-regulatory module is more representative of the “billboard” enhancer model, in which the relative positioning and spacing of transcription factor binding sites is flexible (Spitz and Furlong 2012).

The organization of the 47-bp module revealed a new property of plant developmental enhancers, whereby multiple copies of a *cis*-regulatory element are required in close proximity. Within the fourth 150-bp repeat of *G564*, there are five 10-bp sequences (underlined in Fig. 5e) that have been shown to function as the 10-bp motif when fused to a deletion of the *G564* upstream sequence (Kawashima et al. 2009). The 54-bp fragment contains three of these 10-bp motifs, and all three are required to activate suspensor transcription in the context of the 54-bp fragment. Such homotypic clusters of transcription factor binding sites are a common feature of *cis*-regulatory modules that regulate developmental genes in animals (Lifanov et al. 2003; Cameron and Davidson 2009; Gotea et al. 2010). Our data suggests that homotypic clustering as an organizational principle of developmental *cis*-regulatory modules is conserved not only in animals, but in the plant kingdom as well.

### What transcription factors bind to these suspensor motifs?

We used PLACE (Higo et al. 1999), TRANSFAC (Matys et al. 2003), JASPAR (Mathelier et al. 2014) and the RegSite Databases of Plant Regulatory Elements (http://softberry.com) to check whether any known plant *cis*-elements are present in the 47-bp *G564* suspensor *cis*-regulatory module. No known plant *cis*-elements closely match the 10-bp/10-bp-like/10-bp-related motif or the Region 2 motif sequences. By contrast, the Fifth motif consensus sequence [5′-(A/G)AGTTA-3′] resembles the MYB transcription factor binding site 5′-TAACTG-3′ or, in the reverse strand orientation, 5′-CAGTTA-3′ (Urao et al. 1993; Martin and Paz-Ares 1997). The Scarlet Runner Bean *G564* upstream region can program transcription in the suspensors of transgenic tobacco embryos **(**Figs. 1–4) (Kawashima et al. 2009) and *Arabidopsis* embryos (Kawashima et al. 2009), indicating that the factors binding to these five *cis*-regulatory elements must be spatially localized within suspensors across the plant kingdom. We used a yeast one-hybrid system to show that several *Arabidopsis* MYB transcription factors can bind to the *G564* 54-bp module, supporting the notion that the Fifth motif utilizes a suspensor MYB transcription factor (Henry 2014). The identity of the specific MYB transcription factor binding the Fifth motif, and the transcription factors that interact with the other *G564* suspensor control elements remain to be determined.

### A model for *G564* suspensor transcription

**Fig. 6:**
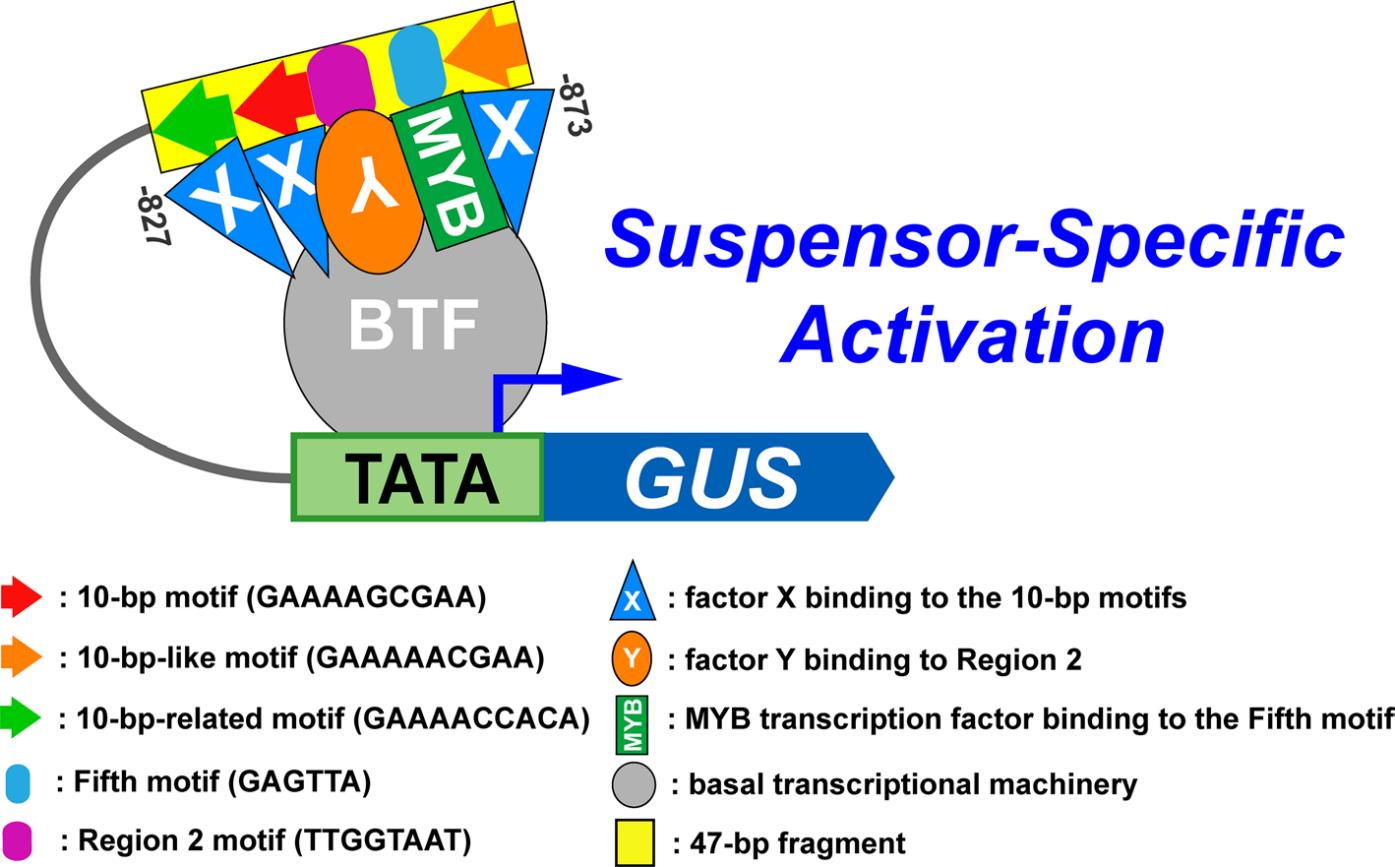
A *G564* suspensor-specific *cis*-control module. *Yellow box* indicates the 47-bp suspensor *cis*-regulatory module (−873 to −827) from the fourth *G564* 150-bp repeat. *Red, orange*, and *green arrows; purple ovals;* and *blue ovals* indicate the 10-bp, 10-bp-like, and 10-bp-related motifs; Region 2 motif; and Fifth motif, respectively. An unknown transcription factor X (*blue triangle*) binds to the 10-bp, 10-bp-like and 10-bp-related motifs. An unknown transcription factor Y (*orange oval*) binds to the Region 2 motif. An unknown MYB-family transcription factor (*green rectangle*) binds to the Fifth motif. The X, Y and MYB transcription factors form a complex to recruit the basal transcriptional machinery that activates *G564* transcription within the suspensor. BTF, basal transcriptional machinery; TATA, RNA polymerase II binding site

How do these five *cis*-elements activate transcription in the suspensor? Figure 6 illustrates a heuristic model for *G564* suspensor transcription by the 47-bp module. This model assumes that the 10-bp, 10-bp-like and 10-bp-related motifs each bind to a transcription factor X because these motifs have been shown to be functionally the same (Kawashima et al. 2009). The Region 2 motif binds a different transcription factor Y. The Fifth motif binds a MYB transcription factor. A notable feature of the 47-bp suspensor control module is the close proximity of all five control elements. This suggests that three copies of transcription factor X, one transcription factor Y and one MYB transcription factor interact to form a compact complex that interacts with the basal transcriptional machinery to activate transcription in the suspensor shortly after fertilization **(**Fig. 6). In order to produce the suspensor-specific expression pattern of *G564*, at least one of these three types of transcription factors must be spatially localized, or active specifically, within the suspensor, and all three types of transcription factors must be present at least as early as the 4-cell embryo, because *G564* is expressed in the two basal cells of the 4-cell embryo (Weterings et al. 2001). The presence of an intact suspensor control module in most of the 150-bp repeats upstream of the *G564* gene might serve to enhance *G564* transcription to a high level in the Scarlet Runner Bean giant suspensor, allowing G564 mRNA to accumulate to high levels **(**Fig. 5b-e) (Weterings et al. 2001).

### Conservation of the suspensor *cis*-regulatory elements in the upstream regions of other suspensor-active genes

Whether the *G564* suspensor *cis*-regulatory elements form part of an extended suspensor-specific gene regulatory network (Peter and Davidson 2015) remains to be determined. A consensus sequence for each of the three types of motif was found in the upstream region of Scarlet Runner Bean genes, such as *GA 20*-*oxidase* and *Wox9*-*like,* which are highly expressed in the suspensor (Le et al. 2007; Le 2013; Henry 2014; Henry and Goldberg 2015). Recently, we showed that the *G564* suspensor control motifs are also required for the region-specific transcription of the *GA 20*-*oxidase* gene (Henry 2014). Thus, the 10-bp/10-bp-like/10-bp-related, Region 2, and Fifth suspensor regulatory sequences are required for the spatially-restricted transcription of at least two Scarlet Runner Bean genes.

What about the functionality of these motifs in the promoters of suspensor-specific genes in other plants, such as *Arabidopsis*? Because the Scarlet Runner Bean *G564* upstream region can program suspensor transcription in several plants, including *Arabidopsis* (Kawashima et al. 2009), we speculate that the *G564* suspensor *cis*-regulatory elements program transcription of genes expressed in the suspensor across the plant kingdom. Although each of these suspensor motifs occur throughout the genome by chance, a cluster of all three types of motif within a few hundred base pairs of sequence is unlikely to occur randomly (Peter and Davidson 2015) and suggests a functional suspensor *cis*-regulatory module. As an example, a specific region of the *Arabidopsis* *WOX8* promoter was shown to activate transcription shortly after fertilization in the zygote and the suspensor of a globular-stage embryo (Ueda et al. 2011), and we found the *G564* suspensor control motifs in this promoter region. Further studies will be required to determine whether the suspensor *cis*-regulatory elements identified in *G564* are also required to program transcription of *WOX8* and other genes in plant suspensor regions.

## Conclusion

To conclude, the data presented here, and elsewhere (Kawashima et al. 2009) identify a suspensor *cis*-regulatory module responsible for the developmental regulation of *G564* expression. The suspensor module contains three types of *cis*-regulatory elements, one of which is required in three copies. Future studies are needed to identify other genes regulated by this module, and the identity of the transcription factors that bind to these *cis*-regulatory elements. With this knowledge, we can begin to build a gene regulatory network specific to the basal cell and suspensor lineage, which is activated shortly after fertilization.

## Electronic supplementary material

Below is the link to the electronic supplementary material.

Online Resource 1 Oligonucleotide sequences for generating *G564* promoter constructs (PDF 63 kb)
