# Supplementary material for: A cis-regulatory module activating transcription in the suspensor contains five cis-regulatory elements
Source: Plant Mol Biol. 2015 Mar 22;88(3):207–17. doi: 10.1007/s11103-015-0308-z (PMC4441743; doi:10.1007/s11103-015-0308-z)
Supplement: Supplementary file 1 — Online Resource 1 Oligonucleotide sequences for generating G564 promoter constructs (PDF 63 kb) [file 11103_2015_308_MOESM1_ESM.pdf]

A *cis*-regulatory module activating transcription in the suspensor contains five *cis*-regulatory elements

Plant Molecular Biology

Kelli F. Henry, Tomokazu Kawashima and Robert B. Goldberg

Robert B. Goldberg (Corresponding author)
Department of Molecular, Cell and Developmental Biology, University of California, Los Angeles, 610 Charles E. Young Dr. East, Los Angeles, CA 90095-7239, USA
Email: bobg@ucla.edu

Online Resource 1 Oligonucleotide sequences for generating constructs

Construct	Forward Oligonucleotides (5' to 3')	Reverse Oligonucleotides (5' to 3')
m54(18-24)	GCTGGAATTCAAGCCTAGAAAAACGAATCTGGCATATTGGTAATGAAAAGCGAA	GATCAATTCCACAGTTTTCGC
m54(18-20)	GCTGGAATTCAAGCCTAGAAAAACGAATCTTTACTATTGGTAATGAAAAGCG	GATCAATTCCACAGTTTTCGC
m54(21-23)	GCTGGAATTCAAGCCTAGAAAAACGAAGAGGGCCTATTGGTAATGAAAAGCGA	GATCAATTCCACAGTTTTCGC
m54(24-26)	GCTGGAATTCAAGCCTAGAAAAACGAAGAGTTAAGCTTGGTAATGAAAAGCGAAG	GATCAATTCCACAGTTTTCGC
m54(27-28)	GCTGGAATTCAAGCCTAGAAAAACGAAGAGTTACTAGGGGTAATGAAAAGCGAAGAA	GATCAATTCCACAGTTTTCGC
m54(27-28)a	GCTGGAATTCAAGCCTAGAAAAACGAAGAGTTACTAAAGGTAATGAAAAGCGAAGAA	GATCAATTCCACAGTTTTCGC
m54(30-31)	GCTGGAATTCAAGCCTAGAAAAACGAAGAGTTACTATTGTGAATGAAAAGCGAAGAAAACC	GATCAATTCCACAGTTTTCGC
m54(30-31)a	GCTGGAATTCAAGCCTAGAAAAACGAAGAGTTACTATTGAAAATGAAAAGCGAAGAAAAC	GATCAATTCCACAGTTTTCGC
m54(32-34)	GCTGGAATTCAAGCCTAGAAAAACGAAGAGTTACTATTGGTCCGGAAAAGCGAAGAAAACCA	GATCAATTCCACAGTTTTCGC
m54(35-37)	TATCCGCTCACAATTCCACA	TAGAGGATCCCCGGGTACCGAGCTCGTGTGGTTTTCTTCGCTTGGAATTACCAATAGTAACTCTTCGTT
m54(38-40)	TATCCGCTCACAATTCCACA	TAGAGGATCCCCGGGTACCGAGCTCGTGTGGTTTTCTTCGAGGTTCATTACCAATAGTAACTCTTCG
m54(41-44)	TATCCGCTCACAATTCCACA	TAGAGGATCCCCGGGTACCGAGCTCGTGTGGTTTTCGGATCTTTTCATTACCAATAGTAACTCT
m54(45-54)	TATCCGCTCACAATTCCACA	TAGAGGATCCCCGGGTACCGAGCTCGGTGTTGGGGATTCGCTTTTCATTACCAAT
47-bp	GCTGGAATTCGAAAAACGAAGAGTTACTATTGG	GATCAATTCCACAGTTTTCGC

Underlined nucleotides are incorporated restriction sites.
Nucleotides shown in blue are mutated relative to the *G564* 54-bp fragment.
